# Regulation of MDM2 E3 ligase-dependent vascular calcification by MSX1/2

**DOI:** 10.1038/s12276-021-00708-6

**Published:** 2021-11-29

**Authors:** Duk-Hwa Kwon, Nakwon Choe, Sera Shin, Juhee Ryu, Nacksung Kim, Gwang Hyeon Eom, Kwang-Il Nam, Hyung Seok Kim, Youngkeun Ahn, Young-Kook Kim, Woo Jin Park, Susan M. Mendrysa, Hyun Kook

**Affiliations:** 1 Department of Pharmacology, Chonnam National University Medical School, Hwasun, Jeollanamdo 58128 Republic of Korea; 2 Basic Research Laboratory for Vascular Remodeling, Chonnam National University Medical School, Hwasun, Jeollanamdo 58128 Republic of Korea; 3 BK21 PLUS Center for Creative Biomedical Scientists, Chonnam National University, Gwangju, South Korea; 4 College of Pharmacy and Research Institute of Pharmaceutical Sciences, Kyungpook National University, Daegu, 41566 Republic of Korea; 5 Department of Anatomy, Chonnam National University Medical School, Hwasun, Jeollanamdo 58128 Republic of Korea; 6 Department of Forensic Medicine, Chonnam National University Medical School, Hwasun, Jeollanamdo 58128 Republic of Korea; 7 Department of Cardiology, Chonnam national University Hospital, Gwangju, 61469 Republic of Korea; 8 Department of Biochemistry, Chonnam National University Medical School, Hwasun, Jeollanamdo 58128 Republic of Korea; 9 College of Life Sciences, Gwangju Institute of Science and Technology, Gwangju, 61005 Republic of Korea; 10 Department of Basic Medical Sciences, College of Veterinary Medicine, Purdue University, West Lafayette, IN 47907 USA

**Keywords:** Disease model, Gene regulation

## Abstract

Vascular calcification increases morbidity and mortality in patients with cardiovascular and renal diseases. Previously, we reported that histone deacetylase 1 prevents vascular calcification, whereas its E3 ligase, mouse double minute 2 homolog (MDM2), induces vascular calcification. In the present study, we identified the upstream regulator of MDM2. By utilizing cellular models and transgenic mice, we confirmed that E3 ligase activity is required for vascular calcification. By promoter analysis, we found that both msh homeobox 1 (Msx1) and msh homeobox 2 (Msx2) bound to the MDM2 promoter region, which resulted in transcriptional activation of MDM2. The expression levels of both Msx1 and Msx2 were increased in mouse models of vascular calcification and in calcified human coronary arteries. Msx1 and Msx2 potentiated vascular calcification in cellular and mouse models in an MDM2-dependent manner. Our results establish a novel role for MSX1/MSX2 in the transcriptional activation of MDM2 and the resultant increase in MDM2 E3 ligase activity during vascular calcification.

## Vascular disease: Driver of deposition of calcium salts identified

The identification of a signaling pathway involved in triggering vascular calcification, the deposition of calcium phosphate crystals in blood vessels, could inform new therapeutic interventions for related cardiovascular complications. Vascular calcification causes significant complications in patients with metabolic syndrome, renal failure, or cardiovascular disease. In their previous work, Hyun Kook and Duk-Hwa Kwon at Chonnam National University Medical School, Jeollanamdo, Republic of Korea, and coworkers demonstrated that the E3 ligase activity of a protein called MDM2 induces calcification. Now, following further mouse trials, the team have identified an upstream signaling pathway involving several development proteins such as MSX1 and MSX2 which activate MDM2. The activation of this signaling axis leads to the degradation of a key protein that would otherwise prevent calcification. The results may provide a platform for novel therapies targeting the condition.

## Introduction

Vascular calcification is caused by abnormal deposition of calcium phosphate crystals in the blood vessels and is linked to cardiovascular morbidity and mortality in patients with metabolic syndrome, chronic renal failure, or atherosclerosis. Although vascular calcification often coexists with other metabolic or cardiovascular diseases, it is currently considered an independent disease process^1^. Moreover, because vascular calcification often results in adverse hemodynamic events owing to reduced blood vessel elasticity and causes increased pulse pressure and systolic hypertension^2^, it is a target for active intervention.

The mechanism of vascular calcification is similar to that of bone mineralization. As does osteogenesis, vascular calcification involves many osteoblast-like cells and intermediates^3,4^. During vascular calcification, contractile vascular smooth muscle cells (VSMCs) undergo a phenotypic switch to osteogenic cells, which results in mineralization of vascular tissue^3,5,6^. Pericytes^7^, adventitial myofibroblasts^8,9^, and vascular progenitor cells^10^ may also be cellular sources of osteoblast-like cells in the development of vascular calcification.

Transdifferentiation into osteoblast-like cells is governed by key osteogenic transcription factors such as msh homeobox 2 (MSX2)^11–13^, runt-related transcription factor 2 (RUNX2, also called core-binding factor a-1, BDFA1), and osterix^14^. As shown by gene mutations in humans^15,16^, MSX2 is expressed in bone and is important for craniofacial, tooth, and limb development^17–19^. It also induces osteogenic differentiation of VSMCs^20,21^ and thereby stimulates vascular calcification^22,23^.

Histone deacetylation mediated by histone deacetylases (HDACs) is associated with diverse cellular events^24^. We previously reported the involvement of HDAC1 in vascular calcification^25^. We observed that either pharmacological inhibition or VSMC-specific genetic ablation of HDAC1 exacerbates vascular calcification. We found that derepression of RUNX2 or other calcifying signaling molecules causes eventual vascular calcification and that the protein level of HDAC1 is significantly reduced in calcified vessels. To elucidate the regulatory mechanism underlying the reduction in HDAC1 in association with vascular calcification, we demonstrated that mouse double minute 2 homolog (MDM2) is upregulated by calcification stress, which results in polyubiquitination-dependent degradation of HDAC1^25^. In the present work, we extend our previous observations of the involvement of MDM2 in vascular calcification by using transgenic mice overexpressing a mutant form of MDM2 as well as mice with VSMC-specific conditional knockout. Importantly, in an effort to identify the upstream regulator of MDM2, we found that MSX1/MSX2-mediated transcriptional activation of MDM2 is critical in the development of vascular calcification. Thus, our findings established a novel MSX1/MSX2-MDM2-HDAC1 signaling pathway in vascular calcification.

## Materials and methods

All experimental procedures were approved by the Chonnam National University Medical School Research Institutional Animal Care and Use Committee and followed the National Institutes of Health *Guide for the Care and Use of Laboratory Animals* (NIH Publication No. 8023, revised 1978).

### Animal models

*Mdm2*^*fl/fl*^ mice were generated as described previously^26^ and maintained on a mixed C57BL/6 and 129/sv genetic background. Smooth muscle (SM)*22α-cre* mice for smooth muscle cell-specific gene deletion were purchased from The Jackson Laboratory (cat. 017491, Bar Harbor, ME, USA) and maintained on a mixed C57BL/6 and 129/sv genetic background. Mice with vascular smooth muscle-specific Mdm2 knockout were obtained by breeding *Mdm2*^*fl/fl*^ mice with *SM22α-cre* mice. Deletion of *MDM2* was confirmed by PCR-based genotyping. *Apolipoprotein E* knockout (*ApoE*^*−/−*^*)* mice were purchased from Jung Ang Animal (Central Lab Animal Inc., Seoul, Korea). The primers used for genotyping were as follows: *Mdm2* floxed—forward, 5′-GTATTGGGCATGTGTTAGACTGG-3′ and reverse, 5′ -CTTCAGATCACTCCCACCTTC; *SM22α cre*—forward, 5′-ATTCTCCCACCGTCAGTACG-3′ and reverse, 5′ -CGTTTTCTGAGCATACCTGGA-3′.

The smooth muscle-22 α promoter fragment (−2615~+223) was inserted into the *pCR2.1* vector using TA cloning (Cosmo Genetech Co. Ltd., Seoul, Korea). Inserts of the *pcDNA6-3xHA-MDM2 WT, pcDNA6-3xHA-MDM2 Y489A*, and *pcDNA6-3xHA-MDM2 ΔR* vectors were subcloned into the *pCR2.1-SM22α* promoter vector using a TOPO™ TA Cloning™ Kit (Cosmo Genetech). Three lines of SM22α promoter-driven MDM2 transgenic mice (TgMDM2 WT, TgMDM2 Y489A, and TgMDM2ΔR) were generated (Macrogen, Seoul, Korea) on the C57BL/6 background in accordance with National Institutes of Health guidelines. Mice were backcrossed for six generations. The level of protein expression in the aortas of transgenic mice was examined by Western blotting with specifically tagged (HA, Sigma) antibodies. Transgenic lines that expressed similar amounts of exogenous proteins were used for further experiments.

All experiments were performed using male mice at 8–9 weeks of age. All animal procedures were reviewed and approved by the Institutional Animal Care and Use Committee of Chonnam National University (CNU IACUC-H-2017-34 and CNU IACUC-HA-2020-17).

### Induction of vascular calcification in vivo

Wild-type C57BL/6 male mice, MDM2 floxed male mice, and MDM2 cKO male mice were used for induction of vascular calcification by administration of vitamin D_3_ as described previously^27^. Vitamin D_3_ (cholecalciferol, 5 × 10^5^ IU kg^−1^ day) in 70 μl of absolute ethanol was mixed with 500 μl of Cremophor (Alkamuls EL-620, Sigma, St. Louis, MO, USA) for 15 min at room temperature and was then combined with 6.2 mL of sterilized water containing 250 mg of dextrose for 15 min at room temperature. Mice were injected subcutaneously with a dose of vitamin D_3_ (150 μl 25 g^−1^, 5 × 10^5^ IU kg^−1^ day) each day for 3 days and maintained for 6 days to induce vascular calcification.

To induce atherosclerotic vascular calcification in *ApoE*^*−/*−^ mice, 8-week-old *ApoE*^*−/−*^ male mice were fed a high-fat/calcium-supplemented diet (TD.02028, Envigo, Indianapolis, USA) for 16 weeks. Calcium deposition in arteries was evaluated by a calcium assay and Alizarin red S staining. All animals were euthanized by intraperitoneal injection of 240 mg kg^−1^ 2,2,2-tribromoethanol anesthesia (T48402, Sigma), and the aortas were isolated.

### Administration of adenoviral MSX1 and MSX2 in mice

For the preparation of purified recombinant adenovirus, AD293 cells were infected with adenoviruses (Ad-GFP, Ad-MSX1, and Ad-MSX2) and were then incubated in a medium containing 10% FBS until a cytopathic effect was observed. The crude virus was purified using cesium chloride (CsCl) gradient centrifugation in an Optima XPN ultracentrifuge (Beckman, Fullerton, CA). Purified recombinant adenovirus was dialyzed with phosphate-buffered saline containing 10% glycerol at 4 °C for 12 h, and adenoviral titers were then determined by using an Adeno-X Rapid Titer Kit (BD Clontech, Laboratories, Inc., Mountain View, CA, USA) following the manufacturer’s instructions. Adenoviruses were diluted with physiological saline in a total volume of 100 μl. Eight-week-old MDM2 floxed mice and MDM2 cKO mice were used in the study. Mice were injected subcutaneously with a low dose of vitamin D_3_ (150 μl 25 g^−1^, 3 × 10^5^ IU kg^−1^ day) each day for 3 days prior to adenovirus injection and were then transduced with adenoviral vectors via tail vein injection of 1 × 10^11^ plaque-forming units (pfu) of recombinant adenoviruses. Six days later, mice were sacrificed and analyzed.

### Human samples

For the human calcification models, both intimal and medial calcification samples were used. Atherosclerosis-associated vascular calcification samples were obtained from autopsied heart patients who died of myocardial infarction. In contrast, medial calcification samples were obtained from autopsied hearts of patients that died of diabetes mellitus complications. For both types of samples, an age-matched normal coronary artery was used as the control. Since these samples were obtained from autopsied tissues, written consent was not required. This study was approved by the institutional review board of Chonnam National University Hospital (CNUH-2018-218) with the exemption of subjects’ written consent. The study conformed to the principles outlined in the Declaration of Helsinki.

### Histology and immunohistochemistry

Tissue samples were fixed with 4% paraformaldehyde and embedded in paraffin. Cross Section (5 μm) were prepared and visualized by Alizarin red S staining and immunohistochemical staining to evaluate vascular calcification and to analyze protein expression, respectively. The following primary antibodies were used for immunostaining: anti-MSX1 (1:100, Thermo Fisher), anti-MSX2 (1:100, Thermo Fisher), anti-RUNX2 (1:100, Thermo Fisher), anti-HA (1:100, Sigma), and anti-His (1:100, Abcam). Micrographs were acquired with an Axio Scan.Z1 scanner (Carl Zeiss Microscopy, GmbH, Jena, Germany) and a laser scanning microscope (DE/LSM700, Carl Zeiss Microscopy).

### Statistical analysis

Statistical significance was analyzed with PASW Statistics 26 (SPSS, IBM Corp, Chicago, IL). For comparisons between two independent groups, a two-tailed unpaired Student’s *t*-test or the nonparametric Mann–Whitney *U*-test was applied after checking for a normal distribution. For comparisons among more than two groups, one-way analysis of variance (ANOVA) or two-way ANOVA with post hoc tests was used depending on the number of main effects. When an interaction between the main effects was confirmed to be significant, stratification was carried out to perform pairwise comparisons. The assumption of equal variance was checked using Levene’s test. Regarding post hoc tests, Tukey’s HSD (honestly significant difference) test was applied for multiple comparisons between data with equal variance, whereas Dunnett’s T3 test was used for data with unequal variance. Significance was determined at the level of *p* < 0.05.

## Results

### Genetic ablation of MDM2 inhibits vascular calcification

**Fig. 1:**
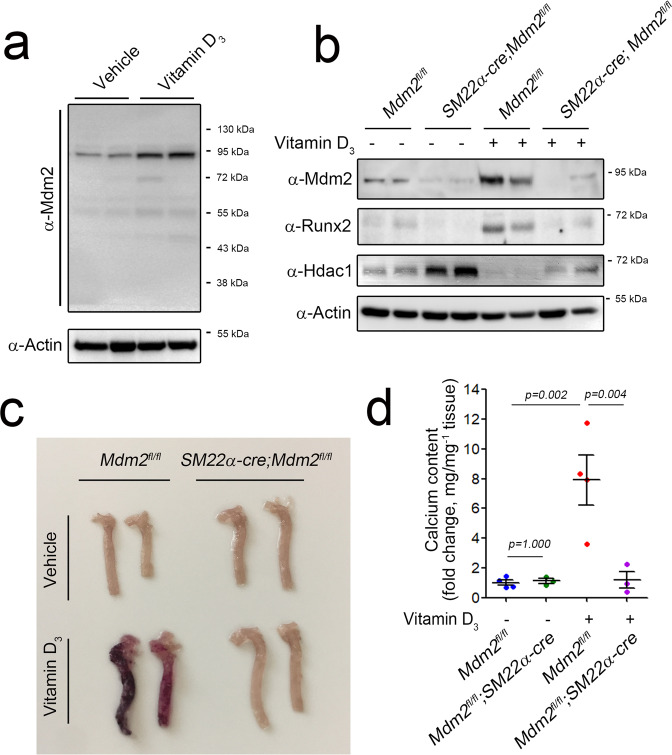
Vitamin D_3_-induced vascular calcification is inhibited in mice with VSMC-specific MDM2 knockout. Vitamin D_3_ (5 × 10^5^ IU kg^−1^) was administered for 3 days, and mice were euthanized on the ninth day. **a** Vitamin D_3_ induced MDM2 expression (90 kDa band) in the mouse aorta. **b** Western blot analysis showing Mdm2, Runx2, and Hdac1 in aortas from *Mdm2*^*fl/fl*^ (wild-type) and *SM22α-cre;Mdm2*^*fl/fl*^ (VSMC-specific *Mdm2* knockout) mice. **c** Aortas were stained with Alizarin red S. **d** Changes in the aortic calcium content. The data were shown as the mean ± s.e.m. values. *P* values for the different comparisons are shown in the figure (*n* = 4).

We first examined the expression of MDM2 in VSMCs and aortas. Inorganic phosphate (Pi) induced both calcium deposition in rat VSMCs (Supplementary Fig. 1a) and the expression of Mdm2 (Supplementary Fig. 1b). Likewise, treatment with either 3 or 5 × 10^5^ IU kg^−1^ vitamin D_3_ induced calcium deposition in blood vessels (Supplementary Fig. 1c). Vitamin D_3_ induced the expression of Mdm2 in mouse aortas (Fig. 1a).

Mice lacking *Mdm2* die early in embryogenesis due to p53-dependent apoptosis^28,29^. Thus, we generated VSMC-specific *Mdm2* knockout mice by breeding *Mdm2*^*fl/fl*^ mice with *SM22α-cre* mice. In *Mdm2*^*fl/fl*^ mouse aortas, vitamin D_3_ significantly increased the expression of MDM2 compared with that in aortas treated with vehicle alone. However, vitamin D_3_ failed to induce MDM2 expression in the aortas of mice with VSMC-specific MDM2 knockout (Fig. 1b).

As visualized by Alizarin red S staining, vitamin D_3_ induced calcium deposition in *Mdm2*^*fl/fl*^ mice but failed to do so in mice with VSMC-specific *Mdm2* knockout (Fig. 1c). Analysis of calcium deposition revealed that vitamin D_3_ induced an increase in the aortic calcium content in *Mdm2*^*fl/fl*^ mice that was abrogated in *SM22α-cre*;*Mdm2*^*fl/fl*^ mice (Fig. 1d). We quantified the level of Runx2 in mouse aortas and found it to be increased in *Mdm2*^*fl/fl*^ mouse aortas but not in *SM22α-cre*;*Mdm2*^*fl/fl*^ mouse aortas (Fig. 1b). Previously, we demonstrated that MDM2-mediated degradation of HDAC1 reverses its transcriptional repression of RUNX2, which causes vascular calcification^25^. Vitamin D_3_ induced dramatic loss of Hdac1 in *Mdm2*^*fl/fl*^ mice but failed to reduce the amount of Hdac1 in the aorta in *SM22α-cre*;*Mdm2*^*fl/fl*^ mice (Fig. 1b).

### Transgenic overexpression of MDM2 is sufficient to initiate vascular calcification in an E3 ligase-dependent manner in vivo

Using pharmacological inhibitors, we previously found that the E3 ligase activity of MDM2 is critical for the degradation of HDAC1 and thereby for the development of vascular calcification^25^. The RING^30^ and HECT^31^ domains are reported to be critical for E3 ligase activity, and Tyr489 is known to be critical for the regulation of the E3 ligase activity of MDM2^32^. Thus, we generated an E3 ligase-dead mutant of MDM2 by substituting Tyr489 with alanine (MDM2 Y489A). We also generated a RING domain deletion mutant (MDM2 ΔR, Supplementary Fig. 2a). Transfection of MDM2 WT significantly reduced the protein level of HDAC1. Transfection of the RING domain deletion mutant, however, failed to do so. The MDM2 Y489A mutation also failed to induce degradation of HDAC1 (Supplementary Fig. 2b). We examined the HDAC1 polyubiquitination activity of MDM2 WT and its mutants. Transfection of MDM2 WT induced smearing of the HDAC1 band, suggesting polyubiquitination of the protein. Polyubiquitination of HDAC1, however, was attenuated when MDM2 Y489A or MDM2 ΔR was transfected (Supplementary Fig. 2c).

**Fig. 2:**
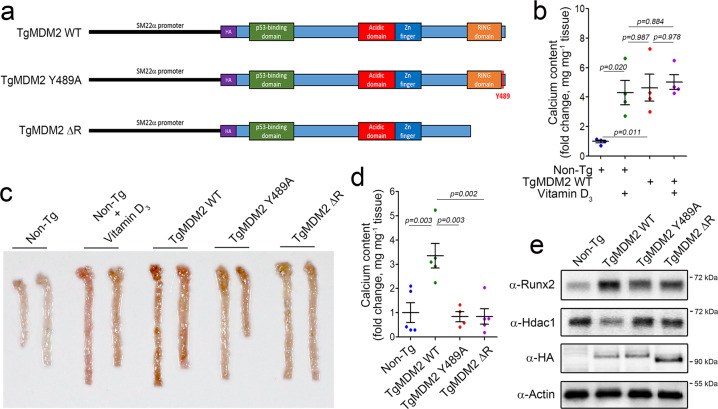
Transgenic overexpression of MDM2 WT but not the MDM2 mutants is sufficient to induce vascular calcification in vivo. **a** Structures of the transgenes used to generate the three transgenic mouse lines. **b** TgMDM2 WT mice expressing wild-type MDM2 in vascular smooth muscle cells showed an increase in calcium deposition similar to that in mice administered vitamin D_3_ (*n* = 4). **c** Alizarin red S staining of whole aortas obtained from TgMDM2 WT, TgMDM2 Y489A, and TgMDM2 ΔR mice. **d** Calcium deposition analysis (*n* = 4–5). **e** Western blot analysis was performed to measure the protein amounts of Runx2 and Hdac1.

To investigate whether the E3 ligase activity of MDM2 is required for the development of vascular calcification in vivo, we generated transgenic mice with overexpression of MDM2 or its mutants in blood vessels by using the *SM22α* promoter. The constructs are shown in Fig. 2a. Overexpression of MDM2 WT itself (third group of dots) was sufficient to induce vascular calcification in vivo, as did vitamin D_3_ (second group of dots). We further investigated whether vitamin D_3_ can potentiate vascular calcification in TgMDM2 WT mice (fourth group). However, it failed to do so (Fig. 2b), suggesting that the effect of vitamin D_3_ on vascular calcification might be saturated because of the high dose of vitamin D_3_ (5 × 10^5^ IU kg^−1^) used.

We next compared the vascular calcification-inducing effect of MDM2 WT with that of the MDM2 mutants. Alizarin red S staining clearly showed vascular calcification in aortas from TgMDM2 WT mice but not in aortas from TgMDM2 Y489A or TgMDM2 ΔR mice (Fig. 2c). Calcium deposition in the aorta was significantly higher in TgMDM2 WT mice than in non-Tg mice. However, the calcium content was not higher in TgMDM2 Y489A or TgMDM2 ΔR mice than in TgMDM2 WT mice (Fig. 2d). Runx2 expression was significantly increased in TgMDM2 WT mice. However, it was not altered in TgMDM2 Y489A or TgMDM2 ΔR mice (Fig. 2e). The amount of Hdac1 protein was reduced in aortas from TgMDM2 WT mice, whereas it was not altered in aortas from TgMDM2 Y489A or TgMDM2 ΔR mice. The expression levels of the MDM2 WT and mutant proteins were almost the same (Fig. 2e).

### Identification of the Pi-responsive element in the MDM2 promoter

How is MDM2 activity controlled? What is the upstream regulator of MDM2? To answer these questions, we first measured the mRNA level of MDM2 and compared it with the eventual increase in the calcium content (Supplementary Fig. 1a). Treatment with Pi for even one day slightly but significantly increased the Mdm2 mRNA level (Supplementary Fig. 3a), and this increase preceded the eventual increase in both the Runx2 mRNA level (Supplementary Fig. 3b) and the calcium content (Supplementary Fig. 1a). These results suggest that MDM2 is primarily regulated in a transcription-dependent manner by calcification stress.

**Fig. 3:**
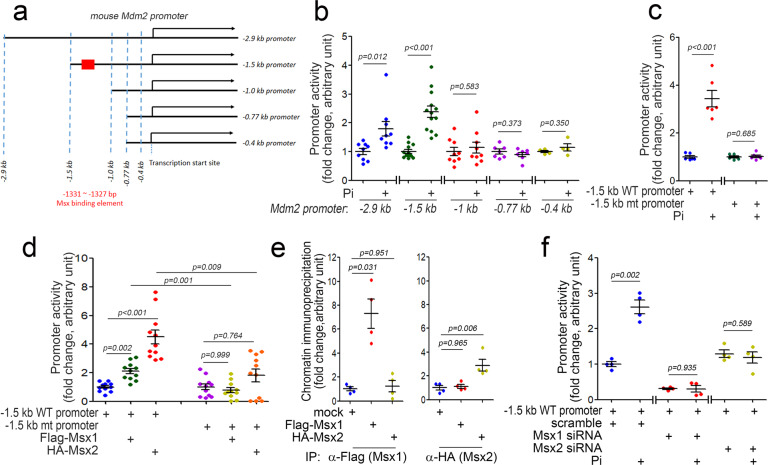
Identification of the Pi-responsive element in the MDM2 promoter. **a** Truncated Mdm2 promoter constructs were used in the study. **b** Pi responsiveness of the truncated Mdm2 promoter activity. Note that Pi increased the activity of both the 1.5-kb and 2.9-kb promoters but not of the 1-kb or shorter promoters. **c** Promoter analysis showing the Pi responsiveness of the Mdm2 promoter with mutation of the MSX binding element (MSXE, −1331 to −1327 bp, shown as a red box in Fig. 3a). **d** Transfection of either Msx1 or Msx2 activated the wild-type MDM2 promoter but failed to activate the MSXE mutant MDM2 promoter. **e** Chromatin immunoprecipitation analysis. **f** Promoter analysis. Pi failed to activate the MDM2 promoter in an Msx1- or Msx2-dependent manner. A10 cells were used.

Next, we generated MDM2 promoter-luciferase constructs containing either a 2.9-kb or 1.5-kb DNA sequence upstream of the transcription start site (Fig. 3a). Pi significantly increased the activity of both the 2.9 kb and 1.5 kb promoters (Fig. 3b). To identify MDM2 activation-specific transcription factors and to demarcate the vascular calcification response element, we performed promoter analysis with shorter truncations of promoter-luciferase constructs with 1.0-, 0.77-, and 0.4-kb promoters (Fig. 3b). In contrast to the 2.9- and 1.5-kb promoters, Pi failed to induce luciferase activity driven by the 1.0-, 0.77-, and 0.4-kb promoters, suggesting that a Pi- responsive element is located between the −1.5 and −1.0 kb regions in the MDM2 promoter.

### MSX1 and MSX2 transcriptionally activate the MDM2 promoter

Which transcription factor(s) are responsible for Pi-induced activation of the MDM2 promoter? Do those transcription factors depend on the Pi-responsive element? To answer these questions, we first analyzed possible transcription factor candidates by a bioinformatics approach (TFsitescan, http://www.ifti.org/cgi-bin/ifti/Tfsitescan.pl). Among the transcription factors identified as potential candidates for binding between the −1.5 and −1.0 kb regions in the MDM2 promoter, we were especially interested in the MSX1/2 binding element with the sequence 3′-AATTG-′5 (in the reverse orientation) spanning the −1331 to −1327 bp region in the MDM2 promoter^33^. MSX1/2 are involved in vascular calcification^34^. However, their function as transcriptional regulators and downstream target molecules in vascular calcification has not been fully elucidated. To confirm whether the −1331 to −1327 bp region is a Pi-responsive element, we used site-directed mutagenesis to mutate the MDM2 promoter (Supplementary Fig. 4a). In contrast to its effects on the MDM2 wild-type promoters, Pi failed to activate the mutant MDM2 promoter with disruption of the −1331 to −1327 bp region-(1.5-kb promoter, Fig. 3c and 2.9-kb promoter, and Supplementary Fig. 4b). The responsiveness of the MDM2 promoter to exogenous MSX1 or MSX2 was also examined; transfection of either Msx1 or Msx2 induced activation of the MDM2 wild-type promoter. However, neither activated the mutant promoter (Fig. 3d). These results suggest that the −1331 to −1327 bp region serves as both a Pi-responsive (PiRE) and an MSX1/2-responsive element (MSXE). Indeed, both exogenous MSX1 and MSX2 are successfully bound to the MSXE (Fig. 3e). We further checked whether Pi-mediated transcriptional activation of the MDM2 promoter is dependent on MSX1 and MSX2. Transfection of Msx1 siRNA inhibited Pi-induced transactivation of the MDM2 wild-type promoter. Likewise, Msx2 siRNA attenuated this transactivation (Fig. 3f).

### The expression of both Msx1 and Msx2 is increased in vascular calcification

**Fig. 4:**
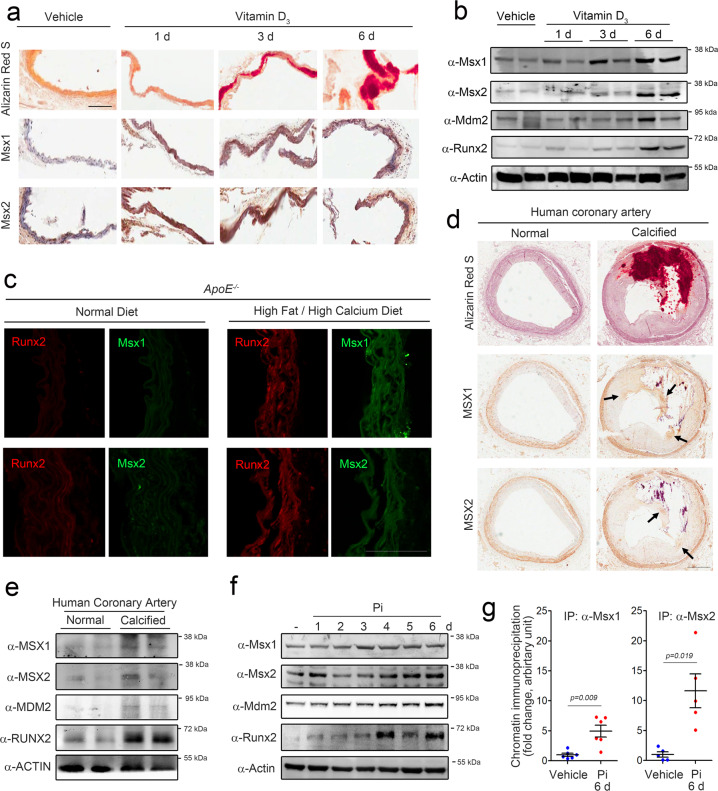
MSX1 and MSX2 expression is induced in calcified vessels. **a** Expression of Msx1 and Msx2 in aortas obtained from vitamin D_3_-treated mice (*n* = 4). Scale bar = 100 μm. **b** Vitamin D_3_ administration increased the protein levels of Msx1 and Msx2. **c** Expression of Msx1 and Msx2 in an alternative vascular calcification model induced in *ApoE*^−*/*−^ mice (*n* = 3) by feeding a high-fat/high-calcium diet for 16 weeks. Scale bar = 100 μm. **d** Immunohistochemical analysis of human calcified coronary artery samples (*n* = 3). Scale bar = 500 μm. **e** Western blot analysis of human coronary artery samples. **f** Pi increased the protein levels of Msx1 and Msx2 in rat VSMCs. **g** Chromatin immunoprecipitation analysis showing the binding of Msx1 and Msx2 to MSXE in the MDM2 promoter in rat VSMCs in the presence of Pi.

Because both MSX1 and MSX2 bound to the MSXE in the MDM2 promoter and because Pi-induced transactivation of the MDM2 promoter was MSX1/2-dependent, we further investigated the roles of MSX1/2 in vascular calcification in our experimental models. It was previously reported that MSX2 is upregulated during vascular calcification^12^. However, considering that MSX1 may share its binding to the MSXE with other proteins containing the same nucleotide sequence and that MSX1 also successfully induces MDM2 transactivation, the role of MSX1 needs to be examined in comparison with that of MSX2. We first checked whether both MSX1 and MSX2 were upregulated in our experimental models. Administration of vitamin D_3_ (5 × 10^5^ IU kg^−1^) to mice successfully induced vascular calcification, as visualized by Alizarin red S staining. The expression levels of Msx1 and Msx2 were increased in these mice (Fig. 4a). The negative control staining without the primary antibody is shown in Supplementary Fig. 5a. Increases in the corresponding protein amounts were also found by Western blot analysis (Fig. 4b).

As we previously reported, chronic administration of a high-fat/high-calcium (high-fat/calcium) diet for 16 weeks in *ApoE*^*−/−*^ mice can induce vascular calcification^25,35^. Compared with that in mice fed the normal diet, the calcium content was increased in mice fed the high-fat/calcium diet (Supplementary Fig. 5b). The high-fat/calcium diet induced an increase in of Runx2 protein expression (red, Fig. 4c). Likewise, both Msx1 and Msx2 were highly expressed in mice fed the high-fat/calcium diet (green, Fig. 4c). The negative control immunofluorescence staining without the primary antibody is shown in Supplementary Fig. 5c. For further investigation in human disease, human calcified coronary artery samples were used for immunohistochemical analysis of MSX1 and MSX2. Compared with normal coronary artery samples, calcified coronary artery samples showed severe intimal calcification with neointimal hyperplasia, as shown by combined Alizarin red S and H&E staining (Fig. 4d). The expression of both MSX1 and MSX2 was increased in the region adjacent to the calcified focus (arrows, Fig. 4d). Western blot analysis of human coronary artery samples further confirmed that the protein levels of both MSX1 and MSX2 were increased in calcified arteries (Fig. 4e).

Next, we sought to determine whether MSX1 and MSX2 can induce vascular calcification in an MDM2-dependent manner and investigated the roles of both MSX1 and MSX2 in the development of vascular calcification. First, as observed in vivo, we determined whether both Msx1 and Msx2 can be upregulated by Pi. We treated rat VSMCs with 2 mM Pi for 6 days. The increase in the calcium content was accompanied by increases in Mdm2 and Runx2 expression (Supplementary Fig. 3a,b). Under the same experimental conditions, the expression of both Msx1 and Msx2 was increased (Supplementary Fig. 6a, b). The Msx2 level increased gradually, and a peak was observed at 5–6 days (Supplementary Fig. 6b), similar to the pattern of Runx2 expression. In contrast, the increase in Msx1 was more prominent at a relatively early stage (1–2 days) of Pi treatment (Supplementary Fig. 6a), suggesting that Msx1 may have distinctive roles in different phases of vascular calcification. Similar patterns of increased expression with phasic differences were observed for the protein expression of Msx1 and Msx2 by Western blot analysis (Fig. 4f). Pi treatment for 6 days induced the binding of endogenous Msx1 and Msx2 to the MSXE in the MDM2 promoter (Fig. 4g and Supplementary Fig. 6c,d).

### Both Msx1 and Msx2 enhance calcium deposition in an MDM2-dependent manner

**Fig. 5:**
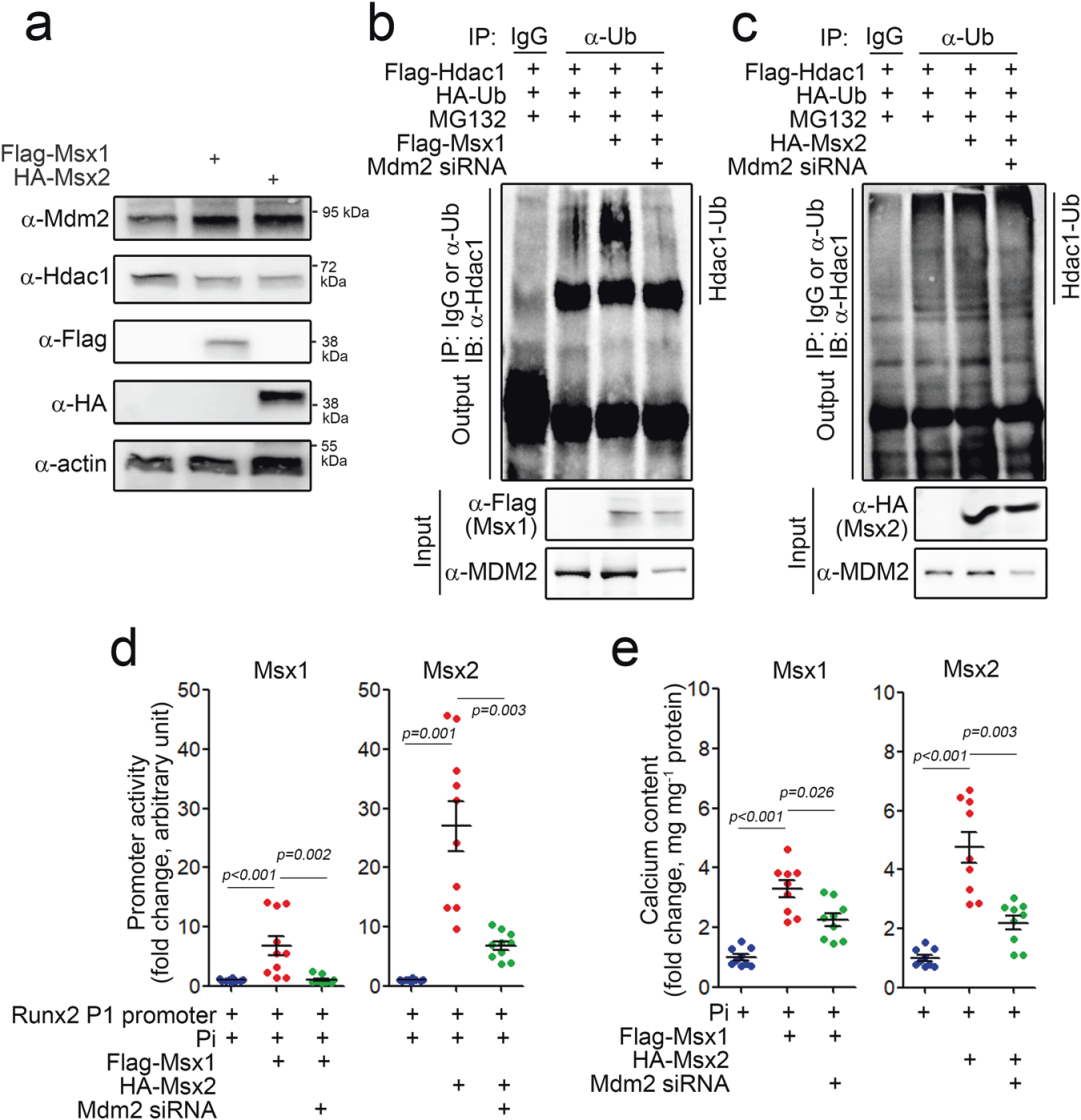
MSX1 and MSX2 induce calcium deposition in VSMCs through the MDM2/HDAC1 pathway. **a** Protein amounts of MDM2 and HDAC1. **b**, **c** Polyubiquitination analysis showing HDAC1 degradation in the presence of Msx1 (**b**) and Msx2 (**c**). **d** Runx2 promoter activity. Msx1- or Msx2-mediated enhancement of Pi-induced promoter activation was attenuated by Mdm2 siRNA. **e** Calcium content. A10 cells were used.

Next, using a cellular model, we investigated whether MSX1 and MSX2 can induce calcium deposition and, if so, whether this induction was MDM2-dependent. Transfection of either *pcDNA3-Flag-Msx1* or *pCS4-HA-Msx2* significantly increased the protein level of Mdm2, whereas it reduced the Hdac1 protein level (Fig. 5a). Msx1 (Supplementary Fig. 7a) and Msx2 (Supplementary Fig. 7b) transfection increased the transcription of *Mdm2* in a dose-dependent manner. Transfection of Msx1 increased the polyubiquitination of Hdac1 (Fig. 5b), which was abolished by simultaneous transfection of Mdm2 siRNA. Exogenous Msx2-induced polyubiquitination of Hdac1 was also attenuated by Mdm2 siRNA transfection (Fig. 5c).

We previously reported that HDAC1 inhibits vascular calcification via transcriptional repression of RUNX2^25^. In this study, we observed that exogenous Msx1 or Msx2 induced Runx2 promoter activity in the presence of Pi, suggesting that the MSX1/2-mediated reduction in HDAC1 expression causes derepression of the Runx2 promoter (Fig. 5d). Exogenous Msx1 or Msx2 potentiated Pi-induced calcium deposition. However, these enhancing effects were blocked by transfection of Mdm2 siRNA (Fig. 5e). These results suggest that both MSX1 and MSX2 can induce calcium deposition by upregulating MDM2, which is followed by downregulation of HDAC1 and derepression of RUNX2.

The effects of MSX1 or MSX2 on the expression of contractile and anti-osteogenic genes were examined. The expression of both SM22α and smooth muscle actin (SMA)^36^ was significantly attenuated by transfection with Msx1 or Msx2 (Supplementary Fig. 8a, b). The anti-osteogenic genes osteoprotegerin (OPG)^37^ and osteopontin (OPN)^38^ were also downregulated by transfection with Msx1 or Msx2 (Supplementary Fig. 8c, d), whereas the expression of the pro-osteogenic gene Runx2 was increased (Supplementary Fig. 8e).

### Crosstalk between Msx1 and Msx2 in calcium deposition

In mice with vascular smooth muscle-specific double knockout of Msx1 and Msx2 on the *LDLR*^*−/−*^ background, Cheng et al. observed a reduction in vascular calcification and suggested that both MSX transcription factors play a redundant role in the induction of vascular calcification^34^. We also observed that similar to MDM2, either MSX1 or MSX2 can enhance vascular calcification in the presence of other calcification stressors, such as Pi, but cannot do so alone. Thus, what is the functional interplay between MSX1 and MSX2 in association with calcium deposition? To answer this question, we first checked whether MSX1 and MSX2 act synergistically to induce vascular calcification.

**Fig. 6:**
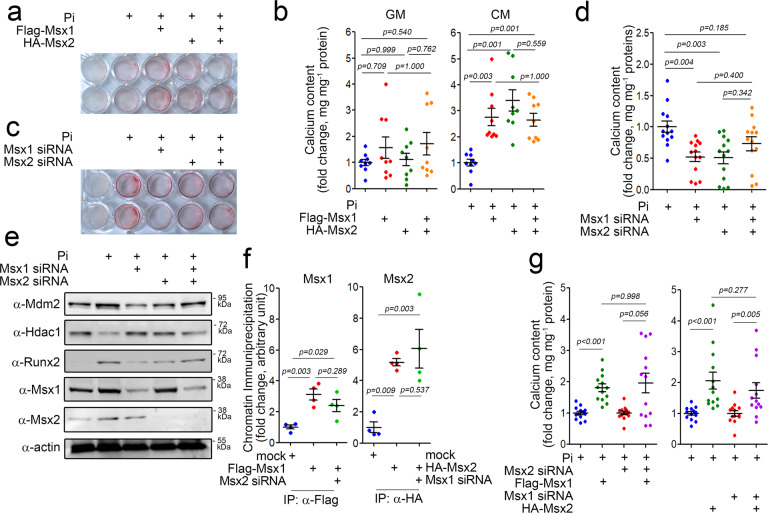
MSX1 and MSX2 redundantly induce calcium deposition. **a** Alizarin red S staining after MSX1 and MSX2 overexpression and Pi treatment. **b** Measurement of the calcium content. **c** Alizarin red S staining after MSX1 and MSX2 knockdown and Pi treatment. **d** Measurement of the calcium content. **e** Western blot analysis. **f** Chromatin immunoprecipitation analysis. Effects of Msx2 knockdown on Msx1 (left panel) and vice versa (right panel). **g** Calcium content. Effects of Msx2 knockdown on Msx1 (left panel) and vice versa (right panel). A10 cells were used.

Pi-induced calcium deposition was enhanced by either Msx1 or Msx2; however, simultaneous treatment with both Msx1 and Msx2 did not enhance the effect of either Msx2 or Msx1 alone (Fig. 6a). The quantitative results are shown in Fig. 6b. In the absence of Pi, both Msx1 and Msx2 alone failed to increase calcium deposition. In addition, cotransfection of Msx1 and Msx2 did not induce calcium deposition. However, in the presence of Pi, either Msx1 or Msx2 alone induced calcium deposition. Interestingly, cotransfection of Msx1 and Msx2 did not potentiate calcium deposition (Fig. 6b). Pi-induced calcium deposition was inhibited by either Msx1 siRNA or Msx2 siRNA. However, no further reduction was observed when both siRNAs were cotransfected (Fig. 6c, d). We further examined the effect of knocking down both Msx1 and Msx2 on their putative downstream targets MDM2 and HDAC1. Treatment with either Msx1 siRNA or Msx2 siRNA attenuated the Pi-induced increase in MDM2 protein expression, which resulted in the replenishment of Hdac1. Cotreatment with both siRNAs did not further reduce the MDM2 protein level. As expected, Hdac1 and Runx2 were reciprocally regulated (Fig. 6e).

We also checked whether Msx1 siRNA or Msx2 siRNA can affect the Pi-induced changes in the expression of contractile and anti-osteogenic genes. Treatment with Pi reduced the expression of SM22a (Supplementary Fig. 9a), SMA (Supplementary Fig. 9b), OPG (Supplementary Fig. 9c), and OPN (Supplementary Fig. 9d). However, these decreases were reversed by transfection of either Msx1 siRNA or Msx2 siRNA (Supplementary Fig. 9a–d). In contrast, the Pi-induced increases in Runx2 and Mdm2 were blocked by Msx1 siRNA or Msx2 siRNA (Supplementary Fig. 9e, f). The changes in the amounts of SM22α and OPN proteins are shown in supplementary Fig. 9g.

It has been reported that MSX1 can form homodimers or heterodimerize with MSX2^39^, suggesting that MSX1 may affect the transcriptional activity of MSX2 and vice versa during vascular calcification. Msx1-induced transactivation of the Mdm2 promoter, however, was not affected by Msx2 siRNA (Supplementary Fig. 10a). Likewise, the effect of Msx2 was not altered by Msx1 siRNA (Supplementary Fig. 10b). Indeed, the binding of Msx1 to the Mdm2 promoter was not altered by Msx2 siRNA and vice versa (Fig. 6f). Likewise, the calcium deposition induced by either Msx1 or Msx2 was not altered by transfection of Msx2 siRNA or Msx1 siRNA (Fig. 6g).

### In vivo adenoviral delivery of the Msx1 or Msx2 gene potentiates vascular calcification in an Mdm2-dependent manner

**Fig. 7:**
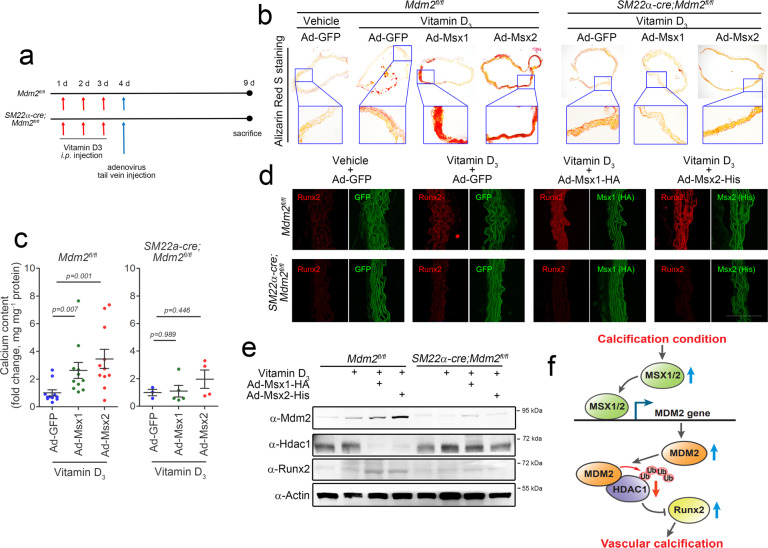
In vivo adenoviral overexpression of either Msx1 or Msx2 potentiates vitamin D_3_-induced vascular calcification in an MDM2-dependent manner in vivo. **a** Timeline for the induction of vascular calcification and adenoviral gene delivery. **b** Alizarin red S staining of aortas. Vascular calcification induced by injection of vitamin D_3_ (3 × 10^5^ IU kg^−1^) was further enhanced by overexpression of either Msx1 or Msx2 in aortas from *Mdm2*^*fl/fl*^ mice. However, this enhancement was not observed in *SM22α-cre;Mdm2*^*fl/fl*^ mice. **c** Calcium content in the aorta (*n* = 3–11). **d** Immunohistochemical analysis with fluorescent antibodies showing the expression of Runx2 in *Mdm2*^*fl/fl*^ (upper panels) and *SM22α-cre;Mdm2*^*fl/fl*^ mice (lower panel). Scale bar = 100 μm. **e** Western blot analysis showing the protein expression of Mdm2, Hdac1, and Runx2. **f** Schematic diagram. Calcification stress induces the expression of the key transcription factors MSX1 and MSX2, which then redundantly activate the transcription of MDM2. MDM2 regulates HDAC1 expression and thereby the RUNX2 protein level in an E3 ligase activity-dependent manner, which results in vascular calcification.

We extended our in vitro results indicating the procalcification effects of MSX1 and MSX2 to our in vivo model. *Mdm2*^*fl/fl*^ and *SM22α-cre;Mdm2*^*fl/fl*^ mice were administered vitamin D_3_ and were then subjected to tail vein injection of Ad-Msx1 or Ad-Msx2 (Fig. 7a and Supplementary Fig. 11). Staining of aorta sections with Alizarin red S revealed that vascular calcification induced by 3 × 10^5^ IU kg^−1^ vitamin D_3_ was enhanced by injection of Ad-Msx1 or Ad-Msx2 (Fig. 7b) in *Mdm2*^*fl/fl*^ mice. In contrast, vitamin D_3_ administration did not have this effect in *SM22α-cre;Mdm2*^*fl/fl*^ mice. Vascular calcification was quantified in those mice. Injection of Ad-Msx1 or Ad-Msx2 significantly increased the calcium content in wild-type mice (Fig. 7c); however, this increase was not observed in knockout mice. The changes in the calcium content were highly related to the changes in Runx2 expression observed by immunohistochemical staining; the expression of Runx2 was enhanced by injection of either Ad-Msx1 or Ad-Msx2, but this enhancement was not observed in knockout mice (Fig. 7d). Mdm2 expression was further increased by the injection of either Ad-Msx1 or Ad-Msx2, whereas the Hdac1 protein level was reduced by injection. Again, these changes were not observed in the aortas of *SM22α-cre;Mdm2*^*fl/fl*^ mice (Fig. 7e). These in vivo results further indicate that both MSX1 and MSX2 induce vascular calcification in an MDM2-dependent manner.

## Discussion

In the current work, we delineated the previously unknown link between MSX transcription factor-mediated activation of MDM2 and vascular calcification. In this signaling pathway, upon calcification stress, the expression of MSX1/2 is increased to transcriptionally activate MDM2 and then induce E3 ligase-dependent degradation of HDAC1, which results in the development of vascular calcification (Fig. 7f). We found that VSMC-specific genetic ablation of MDM2 inhibited vitamin D_3_-induced vascular calcification. VSMC-specific transgenic overexpression of MDM2 WT but not MDM2 Y489A or ΔR induced vascular calcification. Thus, we clearly demonstrated that MDM2 is a potent stimulator of vascular calcification in an E3 ligase-dependent manner. In the present work, transgenic overexpression of MDM2 WT was sufficient to induce calcium deposition in vivo. We also showed that transcriptional regulation of MDM2 is the main effect of MDM2 upregulation. By promoter analysis, we identified a Pi-responsive element in the MDM2 promoter region and found that the MSXE is responsible for both MDM2 promoter activation and calcium deposition.

Apoptosis^40,41^, mitochondrial damage^42^, and perturbation of the endoplasmic reticulum^43^ are important initial stresses in vascular calcification. Similar to bone morphogenic proteins (BMPs) and their downstream SMAD signaling, responses to these vascular calcification stresses converge on several transcription factors, such as RUNX2^44^ and MSX2^45^, resulting in the transdifferentiation of VSMCs or other cells in the adventitia into osteoblast-like cells^46,47^. Indeed, MSX2 is a key transcription factor in vascular calcification^22,23^ and is activated in many signaling pathways, such as the TNF-α^22^, Notch^12^, and BMP2^45^ pathways. In association with metabolic diseases such as diabetes, MSX2 in VSMCs^48^, adventitial myofibroblasts^49^, or the endothelium^21^ activates Wnt signaling, which then results in medial calcification in a paracrine manner.

To direct the fate of progenitor cells into osteoblast-like cells, MSX2 mainly acts as a transcriptional repressor. For example, it inhibits C/EBP-α or PPAR-γ to elicit an antiadipogenic effect^50,51^. MSX2 also directly inhibits myocardin-mediated transactivation of SM22α and other smooth muscle-specific genes to lead VSMCs toward an osteogenic phenotype^23^. However, MSX2 has a dual role to activate the transcription of downstream genes such as heat shock protein 70^52^ or Atoh1^53^. In the current work, our findings suggest that MSX2 induces transcriptional activation of its downstream target, MDM2. Among the MSX transcription factors, MSX2, rather than MSX1, has a key function in the development of atherosclerotic vascular calcification^22,49^. It is noteworthy, however, that MSX1 shares its binding sequence with MSX2 and shows a similar effect on embryonic development, although some mechanistic differences exist^54^. Indeed, both MSX1 and MSX2 are closely involved in neural crest-originated organ formation, such as the formation of craniofacial bone^18,19,55–57^ or cartilage^58^, and in the development of the nervous system^59^ and spinal cord^53^. Interestingly, in the cardiovascular system, both MSX1 and MSX2 participate in the formation of the endocardial cushion and heart valves^60,61^. These proteins interact with T-box proteins to regulate the transcription of connexin 43^62^ and induce phenotypic switching of VSMCs by modulating smooth muscle gene transcription^23^.

MSX1 and MSX2 have redundant roles in the development of major organs^60,61,63^. Likewise, using mice with VSMC-specific double knockout of MSX1 and MSX2, the Towler group clearly showed that both MSX1 and MSX2 redundantly induce atherosclerotic calcification in *LDLR*^*−/−*^ mice fed a high-fat diet^34^. In our study, we unexpectedly observed that MSX1 did not potentiate the effect of MSX2 and vice versa, although there were differences in their expression patterns, for example, in the magnitude of the time window of expression. Likewise, knocking down MSX1 did not affect the effect of MSX2 on calcium deposition, and knocking down MSX2 did not affect the effect of MSX1 on calcium deposition. The finding that knockdown of neither MSX1 nor MSX2 affected the binding of the other factor to the MDM2 promoter suggests that these transcription factors might share a common downstream target.

As a mechanism of medial calcification associated with diabetes under hyperinsulinemic and dyslipidemic conditions, MSX2-expressing adventitial cells in *LDLR*^*−/−*^ mice fed a high-fat diet secrete Wnt1, which induces calcification in the medial layer, as shown by the increase in ALP activity and the eventual calcium deposition^4,49^. However, in the current work, MSX1 and MSX2 were expressed in the muscular medial layer. This spatial change in MSX1/2 expression might be caused by differences in the animal models used (i.e., *ApoE*^*−/−*^ or vitamin D_3_-treated mice). Considering that Pi treatment in VSMCs is sufficient to induce calcium deposition in vitro, an alternative MSX-mediated calcification mechanism in VSMCs or at least in the medial layer may exist.

The initial pathomechanism of intimal calcification seems to be quite different from that of medial calcification in that it is associated with atherosclerosis rather than with metabolic diseases. However, it is noteworthy that both calcification pathways merge into a common mechanism of “osteoblastogenesis-like” transdifferentiation of VSMCs, which can be exemplified by RUNX2. We also observed that the MSX1/2-MDM2 pathway is similarly strongly involved under both calcification conditions, suggesting that this signaling cascade may contribute to the common mechanism of calcification.

In the current work, we established a link between MSX1/2 and MDM2-mediated derepression of HDAC1. We further confirmed that the E3 ligase activity of MDM2 is indispensable for the induction of vascular calcification. Thus, this signaling pathway may provide a novel platform for the development of therapeutics for the deleterious cardiovascular results of vascular calcification.

## Supplementary information

Supplementary information
