# Supplementary material for: Regulation of MDM2 E3 ligase-dependent vascular calcification by MSX1/2
Source: Exp Mol Med. 2021 Nov 29;53(11):1781–91. doi: 10.1038/s12276-021-00708-6 (PMC8639964; doi:10.1038/s12276-021-00708-6)
Supplement: Supplementary file 1 — Supplementary information [file 12276_2021_708_MOESM1_ESM.pdf]

Regulation of MDM2 E3 ligase-dependent vascular calcification by MSX1/2

Duk-Hwa Kwon[1,2,3], Nakwon Choe[1,2], Sera Shin[1,2], Juhee Ryu[2,4], Nacksung Kim[1,3], Gwang Hyeon Eom[1,3], Kwang-Il Nam[5], Hyung Seok Kim[3,6], Youngkeun Ahn[2,7], Young-Kook Kim[2,3,8], Woo Jin Park[2,9], Susan M. Mendrysa[10], Hyun Kook[1,2,3]

Supplementary Information

Supplementary Material and Methods

Reagents

Antibodies against anti-MDM2 (1:1000, ab16895), RUNX2 (1:1000, ab23981), and p-S166 MDM2 (1:1000, ab131355) and SM22 α (1:1000, ab10135) were from Abcam (Abcam, Cambridge, UK); Flag (1:1000, F7425 and F1804), HA (1:1000, H9658), and Actin (1:1000, A2066) were from Sigma (Sigma-Aldrich Corp., St. Louis, MO, USA); HDAC1 (1:1000, sc7872), MSX1 (1:1000, sc517211), MSX2 (1:1000, sc393986), Ub (1:1000, sc8017), His (1:1000, sc8036), OPN (1:500, sc73631) and Gapdh (1:1000, sc16574) were from Santa Cruz Biotechnology (Santa Cruz Biotechnology, Inc., Santa Cruz, CA, USA); MSX1 (1:1000, PA5-23176) and MSX2 (1:1000, PA5-41436) were from ThermoFisher (Thermo Fisher Scientific Inc., Rockford, IL, USA). Anti-mouse (1:5000, 7076S) or anti-rabbit (1:5000, 7074S) IgG peroxidase-conjugated secondary antibodies were purchased from Sigma (Sigma-Aldrich Corp., St. Louis, MO, USA). Alexa Fluor 488-conjugated secondary antibodies (1:200, A11001 and 1:200, A11008) and Alexa 568-conjugated secondary antibodies (1:200, A11011 and 1:200, A11004) were purchased from Invitrogen.

Adenoviral MSX1 (111502A) and MSX2 (111509A) were purchased from Applied Biological Materials Inc (ABM Inc., Richmond, BC, Canada). Cholecalciferol (vitamin D_3) and 2,2,2-tribromoethanol were purchased from Sigma (Sigma-Aldrich Corp., St. Louis, MO, USA). MG132 was purchased from Calbiochem (EMD Millipore). 3,3'-Diaminobenzidine (DAB) substrate and ABC-HRP kits were purchased from Vector (Vector laboratories Inc.,

Burlingame, CA, USA). *MSX1* siRNA, *MSX2* siRNA, *MDM2* siRNA, and scramble were purchased from Bioneer (Daejeon, Korea).

Plasmids

Flag MSX1 was generously provided by Prof. HanSol Lee (Inha University, Incheon, Korea). *HA-MSX2* was kindly provided by Prof. Kwang-Youl Lee (Chonnam National University, Gwangju, Korea). *pBJ5.1-Flag-HDAC1*, *pcDNA3-HA-Ub*, *pcDNA6-3xHA-MDM2 WT*, and *pcDNA6-3xHA-MDM2ΔR* were described previously[1]. *pcDNA6-3xHA-MDM2 Y489A* was constructed by site-directed mutagenesis (CosmoGeneTech, Seoul, Korea) based on *pcDNA6-3xHA-MDM2 WT.* Full-length wild-type (-2.9 kb) and various lengths of MDM2 promoter luciferase plasmid were subcloned into pGL3- basic vector (Bionics corp., Seoul, Korea). MSX binding element (MSXE)-disrupted MDM2 promoter luciferase was constructed by Bionics (Bionics). All plasmids were checked before use by direct sequencing.

Cell cultures

Rat vascular smooth muscle cells (RVSMCs) were isolated from thoracic aorta of 6~8-week-old Sprague-Dawley male rats euthanized by anesthesia with 240 mg/kg 2,2,2-tribromoethanol (T48402, Sigma, St. Louis, MO, USA). The aortas were washed with ice-cold 1xPBS and incubated with 1 mL 0.2% collagenase I solution (LS004196, Worthington Biochemical Corp., Lakewood, NJ, USA) in Ham's F12 medium (12-615F, Lonza Group Ltd., Basel, Switzerland) containing antibiotics (300 Uml^{-1} penicillin, 300 U ml^{-1} streptomycin) at 37℃ for 30 min. The aorta was opened lengthwise, and the intima was scraped on the

luminal surface. Tissue samples were minced in dissection medium with Ham's F12 media containing antibiotics and the dissected tissues were incubated in 0.2% collagenase I solution at 37°C overnight with shaking. The dissected tissues were attached to 100-mm dishes and cultured in 10% fetal bovine serum (FBS; GIBCO) in DMEM (GIBCO) with antibiotics. RVSMCs were used at passages 2 to 7. A10 cells derived from embryonic rat aorta were purchased from American Type Culture Collection (ATCC, CRL-1476, Manassas, VA, USA) and were maintained in 10% FBS in DMEM with antibiotics. Human embryonic kidney 293T cells were obtained from the Seoul Korean Cell Line Bank (KCLB, 21573, Seoul, Korea) and were maintained in 10% FBS in DMEM with antibiotics. All cells were incubated in a humidified atmosphere with 5% CO_2.

Induction of vascular calcification in vitro

For the induction of vascular calcification in VSMCs, the cells cultured in growth medium were changed with calcification medium containing 2 mM or 4 mM inorganic phosphate (pH 7.4) for up to 3 days or 6 days. The medium was changed every 2 days. Calcium deposition in VSMCs was determined after washing with 1x PBS twice.

Quantification of calcium deposition

Cells and tissues were decalcified with 0.6N HCl at 4°C for 24 hours. The calcium content of the HCl supernatants was determined colorimetrically using the QuantiChrom calcium assay kit (QuantiChromTM Calcium Assay Kit, BioAssay Systems, Hayward, USA) according to the manufacturer's instructions. Briefly, 5 μL of the samples was transferred to a 96-well

plate and 200 μl working reagent was added (1:1, solution A and B). Mixed samples were briefly incubated and absorbance was measured at 570 nm by using an ELx808 absorbance reader (BTELX808, BioTek Instruments, Winooski, VT, USA). After decalcification, cells were washed 3 times with 1xPBS and lysed with 0.1N NaOH/0/1% SDS to extract proteins. The calcium content of cells was then normalized to the total protein amount, whereas that of the tissues was normalized to tissue dry weight.

Alizarin Red S staining

The cells were washed with 1xPBS and fixed with 10% formalin for 30 min at RT. After being washed three time with distilled water, the cells were stained with 40 mM alizarin red S solution (pH 4.2, Sigma-Aldrich) for 1 hour at room temperature and washed with 1xPBS to remove nonspecific staining.

To determine arterial calcification, the aorta was collected and stored in 70% ethanol. The arterial tissue sample was placed in 10 mL of alizarin working solution containing 0.8% Alizarin red S in 0.5% KOH for 24 h with rotation and then switched to 10 ml of 0.05% KOH for 24 h to remove nonspecific stain from the tissues.

Total RNA preparation and Quantitative Real-time PCR

Total RNA was extracted using TRIzol Reagent (Invitrogen, Waltham, MA, USA) according to the manufacturer’s instructions. The cDNA synthesis was generated using iScript™ cDNA Synthesis Kit (Bio-Rad Laboratories, Inc., Hercules, CA, USA) and analyzed by quantitative

real-time PCR using a QuantiTech SYBR Green RT- PCR Master Mix (QIAGEN, Valencia, CA, USA) and a Rotor gene Q (Qiagen, Hilden, Germany). All data were normalized to GAPDH. Relative mRNA levels were quantified using the $2^{-\Delta\Delta Ct}$ method. To rule out possible genomic DNA contamination, primers were designed to include an intervening intron. Primers for quantitative real-time polymerase chain reaction (qRT-PCR) were as follows: mouse MDM2, forward, 5’-GAG AAC ACA GAT GAG CTA CC-3’, and reverse, 5’-CCAGGCAATCACCAGAAT-3’; rat MDM2, forward, 5’-GCG AGC GGA GAC GGA CAC AC-3’, and reverse, 5’-GGG CTC TGT GGC GCT TCC TC-3’; mouse RUNX2, forward, 5’-CCC AGC CAC CTT TAC CTA CA-3’, and reverse, 5’-CAG CGT CAA CAC CAT CAT TC-3’; mouse GAPDH, forward, 5’-GCA TGG CCT TCC GTG TTC CT-3’, and reverse, 5’-CCC TGT TGC TGT AGC CGT ATT CAT-3’; rat SM22α, forward, 5’-CTT GAA GGC AGC TGA GGA TTA T-3’, and reverse, 5’-CAA ACT GCC CAA AGC CAT TAC-3’ ; rat Smooth muscle α actin, forward, 5’-AGG GAG TGA TGG TTG GAA TG-3’, and reverse, 5’- GGT GAT GAT GCC GTG TTC TA -3’ ; rat OPG, forward, GGC AGG GCA TAC TTC CTG TTG CC, and reverse, 5’-TCG GTT GTG GGT GCG GTT GC-3’; rat OPN, forward, 5’-CCG TGA GGC CGC AGT TCT CC-3’, and reverse 5’-CAG AGG GCA CGC TCA GAC GC-3’ ; rat GAPDH, forward, 5’-TGC ACC ACC AAC TGC TTA G-3’, and reverse, 5’-GAT GCA GGG ATG ATG TTC-3’. Primers for mouse MSX1 (Mm00440330_m1), rat MSX1 (Rn00667535_m1), mouse MSX2 (Mm00442992), rat MSX2 (Rn01448617_m1), and rat RUNX2 (Rn01512298_m1) were pre-designed qPCR primers that were purchased from ThermoFisher.

Immunoprecipitation and Western blot analysis

Cells and tissues were lysed with 0.5% NP-40 solution including 50 mM Tris (pH 8.0), 150

mM NaCl, 1 mM EDTA, 1 mM DTT, 1 mM phenylmethylsulfonyl fluoride, 1 mM Na_3VO_4, and 1 μg/mL each of leupeptin, pepstatin, and aprotinin. One milligram of proteins was then immunoprecipitated overnight at 4 °C with the indicated antibody. After extensive washing with lysis buffer, the immunocomplexes were analyzed by western blotting assay. The lysates were separated by SDS-PAGE and transferred overnight at 120 mA onto a PVDF (Millipore, Bedford, MA, USA) and blocked with 5% skim milk in 1x TBST. Membranes were incubated with the specific primary antibodies overnight at 4°C. After 3 washes in 1x TBST, membranes were incubated with horseradish peroxidase-linked secondary antibodies for 1 hour at room temperature. Membranes were again washed 3 times in 1x TBST, and protein bands were visualized by enhanced chemiluminescence using Western Blotting Luminol Reagent (Santa Cruz) and a Fusion FX7 imaging system (Vilber, Marne-la-vallée, France).

Promoter luciferase reporter assay and MTT assay

For the luciferase assay, A10 cells were plated in 24-well plates and transiently transfected with plasmids containing pCMV-beta-galactosidase using Lipofectamine (ThermoFisher) and Lipofectamine RNAiMAX (ThermoFisher) according to the manufacturers' protocols. The cells were treated with 4 mM Pi for 3 days. The luciferase activity was measured by using the Luciferase Assay System (E1500, Promega) and normalized to β-galactosidase activity. The data are representative of at least two to three independent experiments.

The cells were plated in 96-well plates and transiently transfected with plasmids. After 48 hours, the cells were treated with 10 μL 3-(4,5-dimethylthiazol-2-yl)-2,5-diphenyltetrazolium bromide (MTT, Sigma) and incubated for 2 hours at 37℃ and then changed to 100 μL

dimethyl sulfoxide (DMSO). Absorbance was measured at 570 nm by using an ELx808 absorbance reader (BioTek Instruments).

Chromatin immunoprecipitation (ChIP)

Chromatin immunoprecipitation assays were performed by using an EpiQuik chromatin immunoprecipitation kit (EpiGentek, Farmingdale, NY, USA) according to the manufacturer's instructions. Briefly, A10 cells were treated with 1% formaldehyde for 10 min for cross-linking between proteins and DNA and sonicated to shear the chromatin into fragments of < 500 bp. The chromatin complex was immunoprecipitated with indicated primary antibody, while the negative control was immunoprecipitated with nonimmunized IgG. Primers for qPCR of rat MDM2 promoter flanking the MSXE were forward, 5′-TGC TCT TAA TCT CTG AGC CAT CTC-3′, and reverse, 5′-CAG ATT AAC ACA GAG GTC ACC TAC-3′.

Supplementary Figures

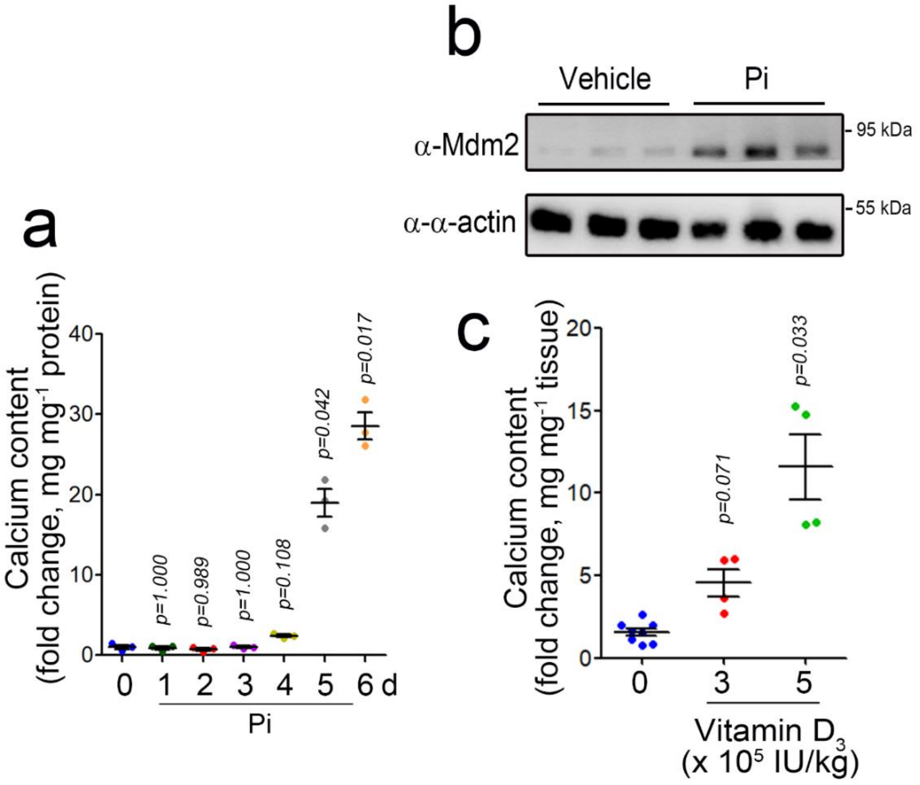

Supplementary Fig. 1. Calcium deposition by inorganic phosphate (Pi) in VSMCs and vitamin D_3 administration in mice. (**a**) Pi induces calcium deposition in VSMCs. Eventual increase in calcium content takes place by the 4th day. (**b**) Mdm2 protein expression in Pi-treated VSMCs. (**c**) Vitamin D_3 administration induces calcium deposition in the aorta.

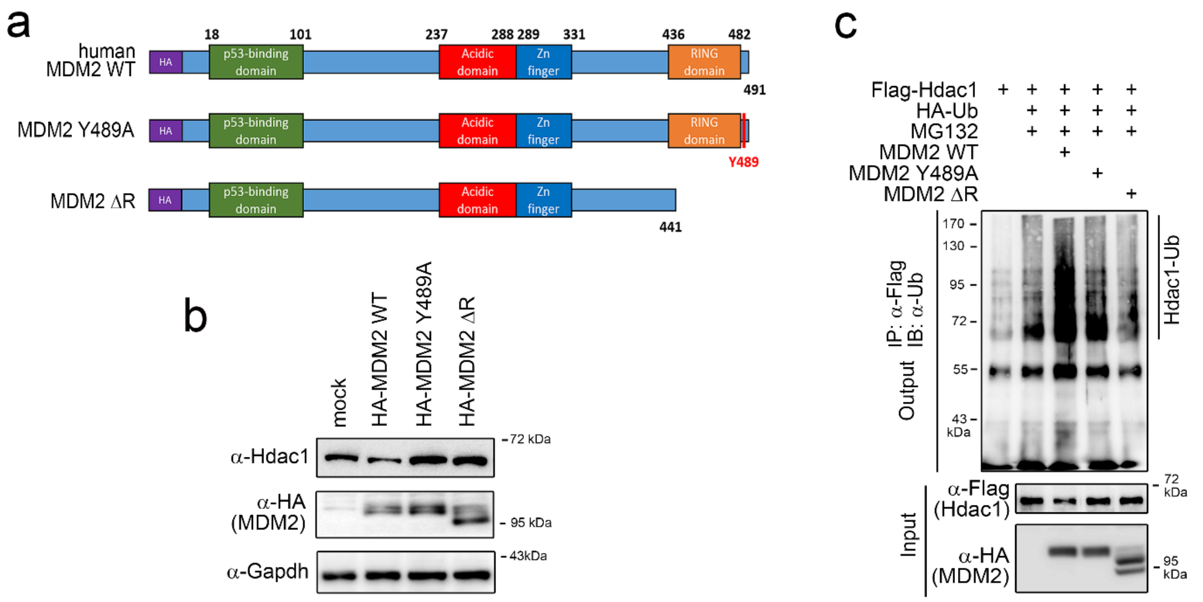

Supplementary Fig. 2. MDM2 WT, MDM2 Y489A, MDM2 ΔR and HDAC1 ubiquitination. (**a**) Schematics of the human MDM2 wild-type (WT), MDM2 Y489A, and MDM2 ΔR constructs used in the study. (**b**) Changes in the amount of HDAC1 in A10 cells. Mutant MDM2 failed to degrade HDAC1. (**c**) Ubiquitination assay.

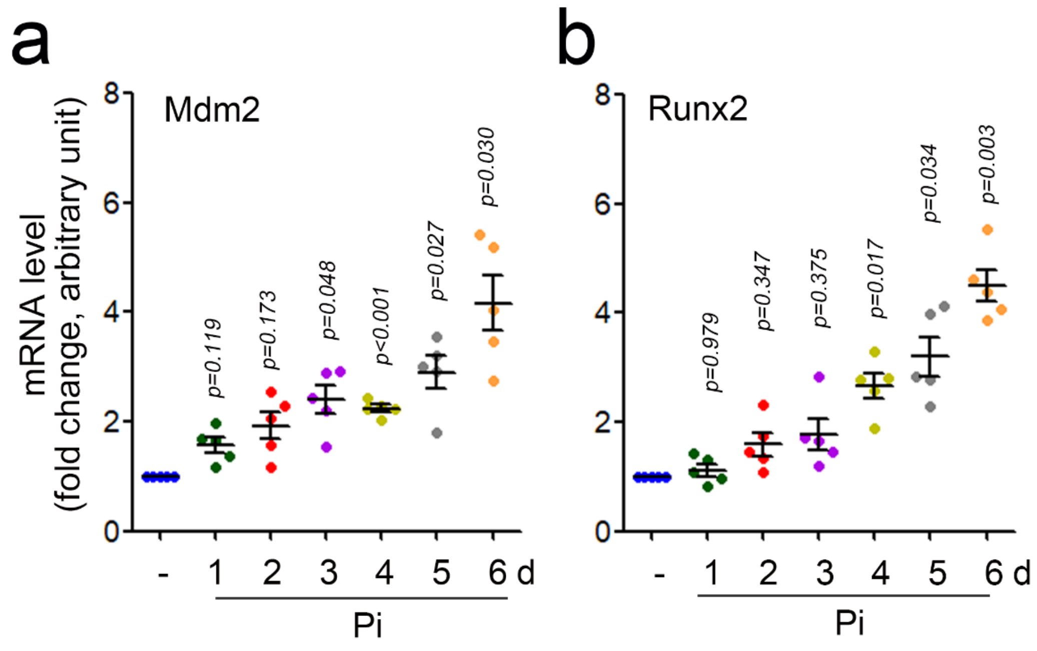

Supplementary Fig. 3. Mdm2 mRNA increase by Pi treatment. (**a**) Increase in Mdm2 mRNA by Pi treatment in VSMCs. (**b**) Increase in Runx2 mRNA by Pi treatment.

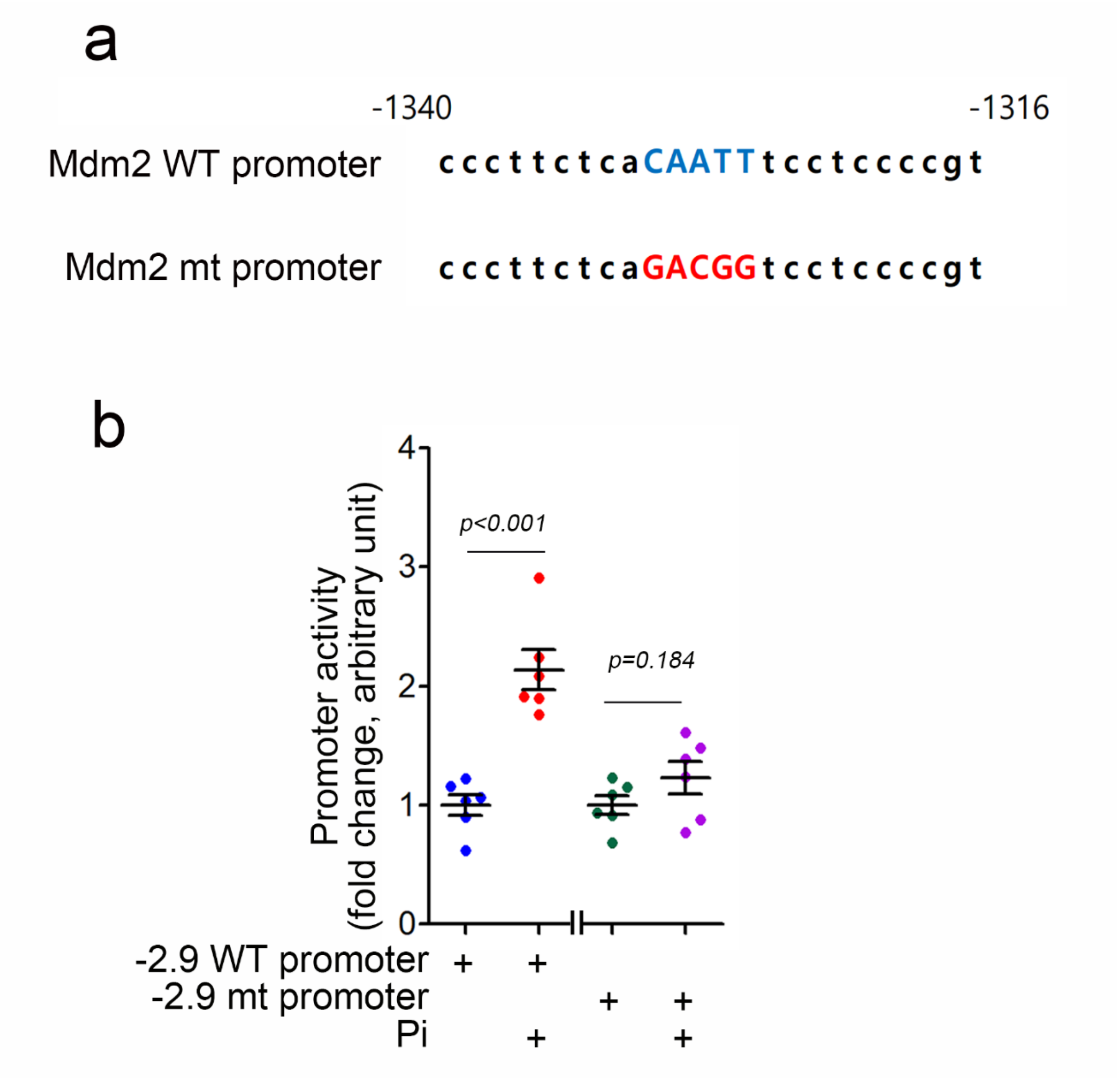

Supplementary Fig. 4. MSX1/2-binding element (MSXE) in Mdm2 promoter and Pi-responsiveness of its mutant. (**a**) Sequence alignment of Mdm2 wild-type promoter and its mutant that was used in the current study. (**b**) Pi-responsiveness of -2.9kb Mdm2 wild-type promoter and its mutant.

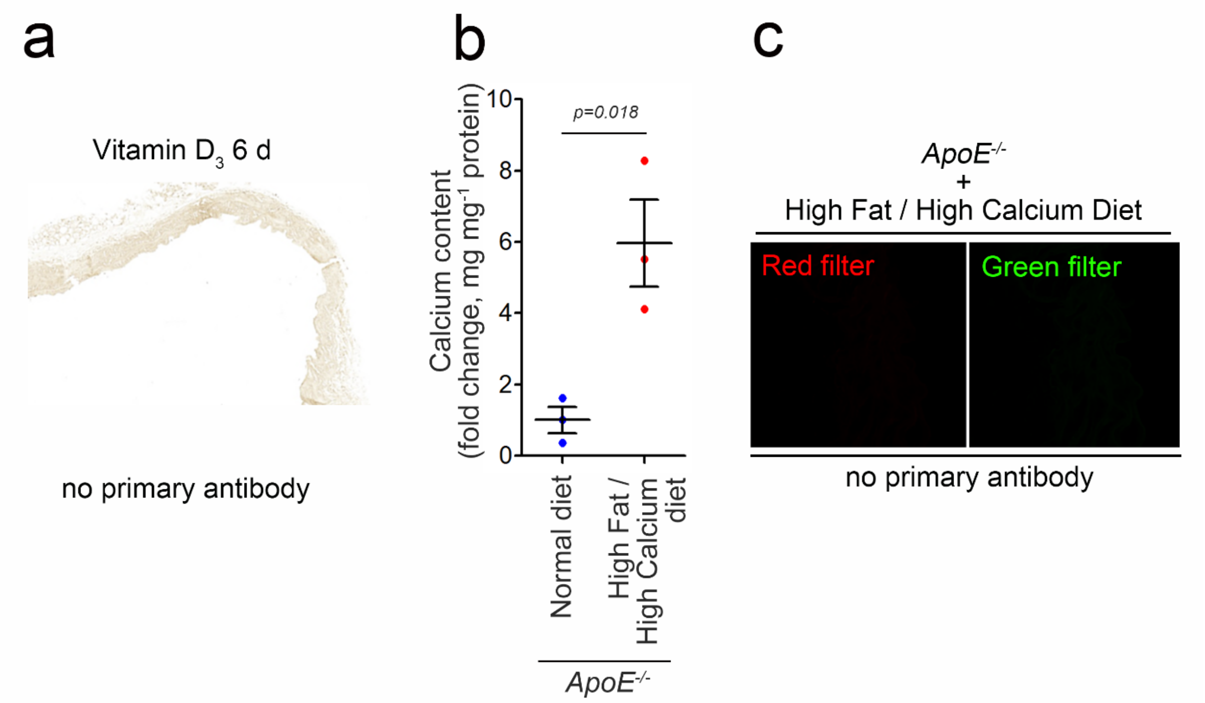

Supplementary Fig. 5. High fat plus high calcium diet-induced vascular calcification in mice. (**a**) Negative control image of immunohistochemical analysis. The same experimental condition as Figure 4a right-most panels (vitamin D_3 6 days) except for the absence of primary antibody. (**b**) High fat plus high calcium diet increased the calcium deposition in the *ApoE*$^{-/-}$ mouse aorta. (**c**) Negative control image of fluorescent immunohistochemical analysis. The same experimental condition as Figure. 4c right panels (high fat plus high calcium diet) except for the absence of primary antibody.

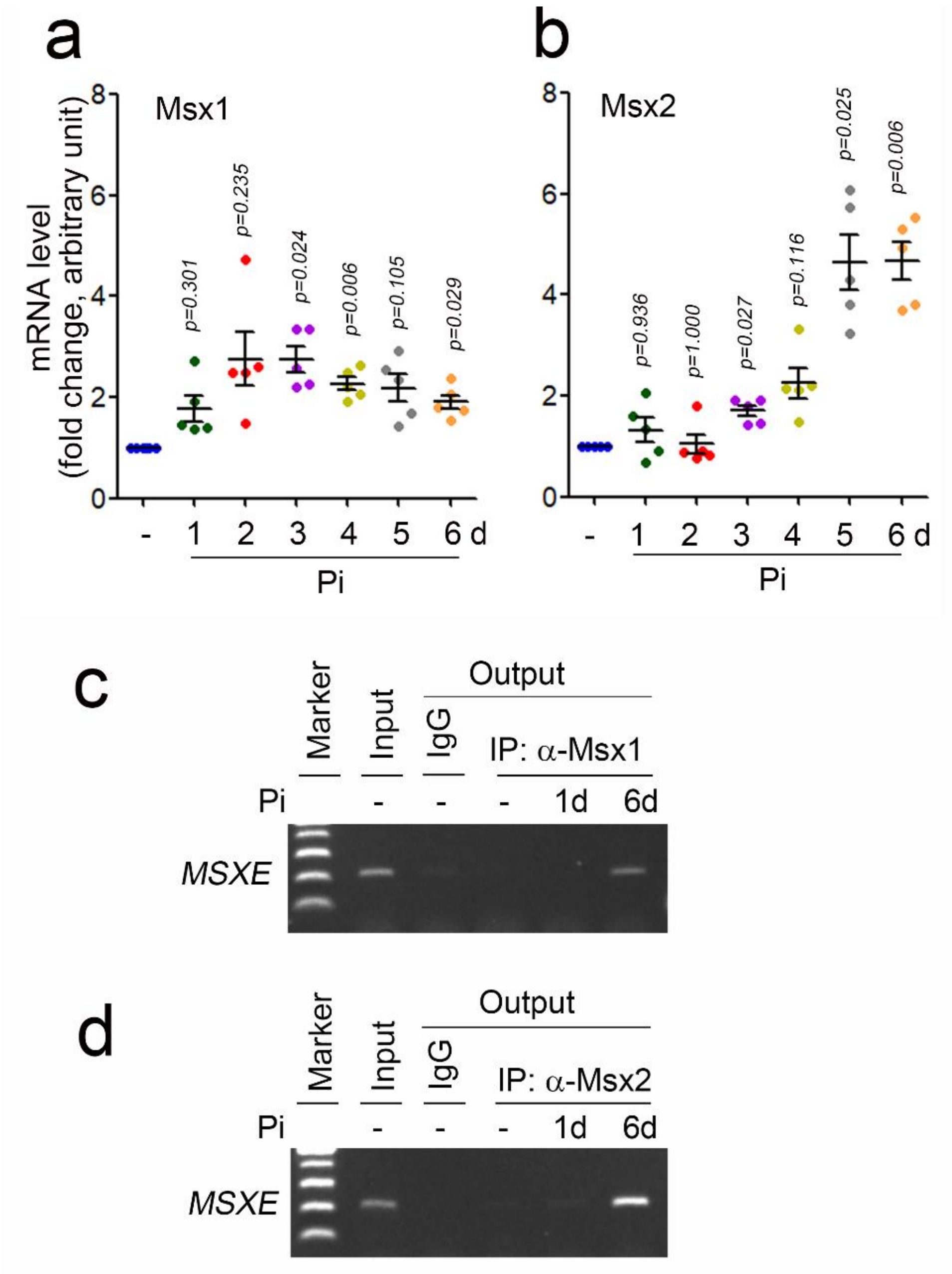

Supplementary Fig. 6. Changes in Msx1 and Msx2 mRNA levels in Pi-treated VSMCs. (**a**) qRT-PCR results of change in Msx1 mRNA levels. (**b**) Msx2 mRNA levels. (**c-d**) Representative gel pictures of chromatin immunoprecipitation analysis to show the binding of endogenous Msx1 (**c**) and Msx2 (**d**) to Mdm2 promoter in the presence of Pi in VSMCs.

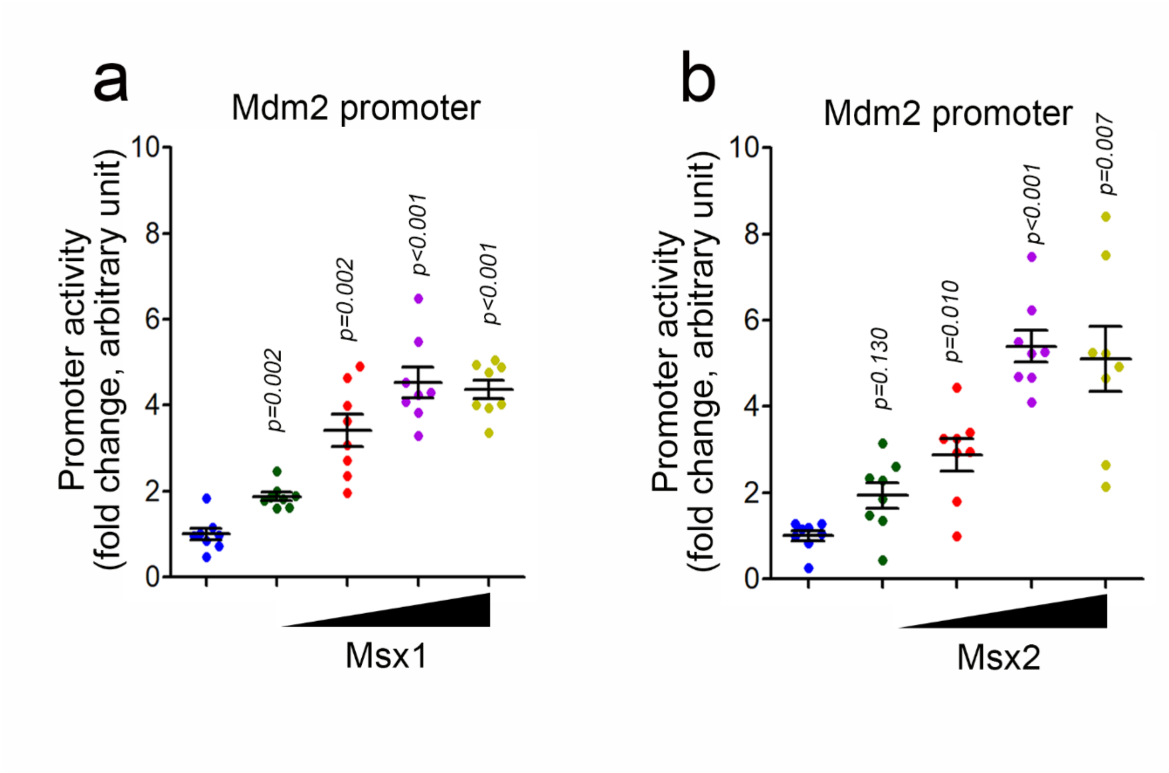

Supplementary Fig. 7. Effects of transfection of Msx1 and Msx2 on Mdm2 promoter activity. (**a-b**), Msx1 (**a**) and Msx2 (**b**) increased Mdm2 promoter activity in a dose-dependent fashion.

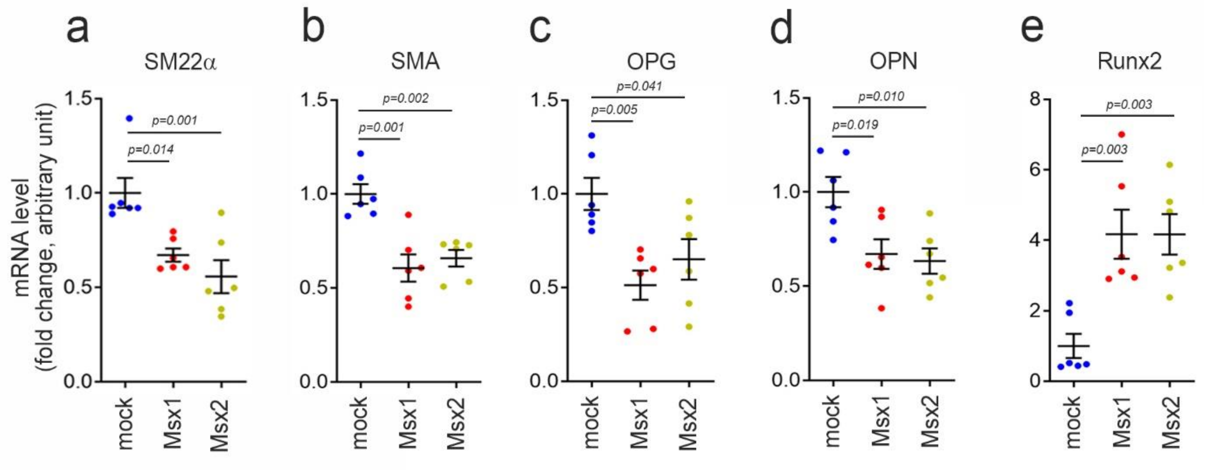

Supplementary Fig. 8. Effects of transfection of Msx1 and Msx2 on VSMC contractile genes or anti-osteogenic genes. VSMC contractile genes (**a-b**) Contractile genes (**a**) Smooth muscle 22 α, SM22α (**b**) smooth muscle actin, SMA (**c-d**) Anti-osteogenic genes (**c**) Osteoprotegerin, OPG (**d**) Osteopontin, OPN, (**e**) Pro-osteogenic gene, RUNX2.

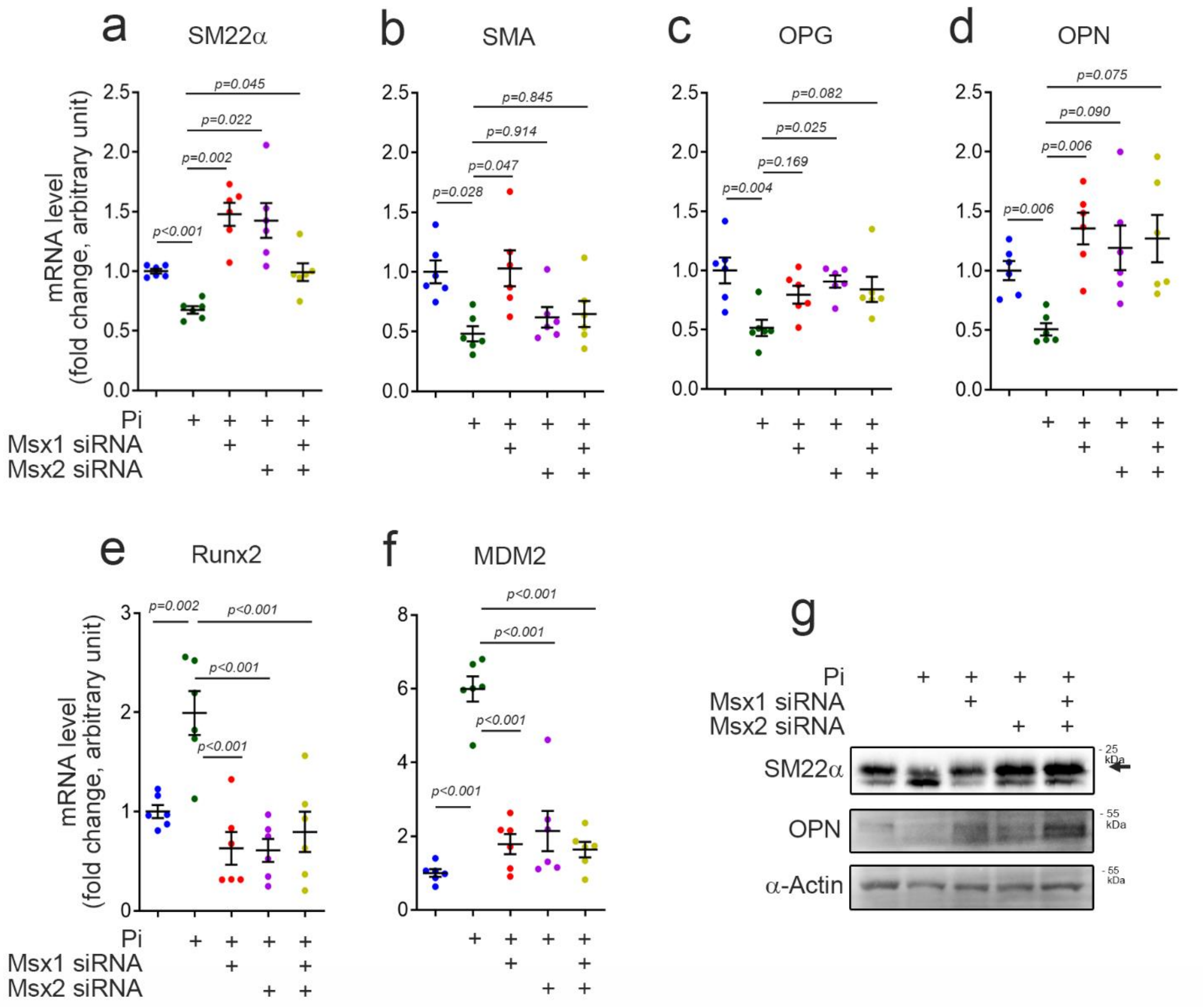

Supplementary Fig. 9. Effects of transfection Msx1 siRNA or Msx2 siRNA on Pi-induced changes in expressions genes. (**a-b**) Contractile genes (**a**) Smooth muscle 22 α, SM22α (**b**) smooth muscle actin, SMA (**c-d**) Anti-osteogenic genes (**c**) Osteoprotegerin, OPG (**d**) Osteopontin, OPN, (**e-f**) Pro-osteogenic genes, RUNX2 and MDM2. (**g**) Western blot analysis

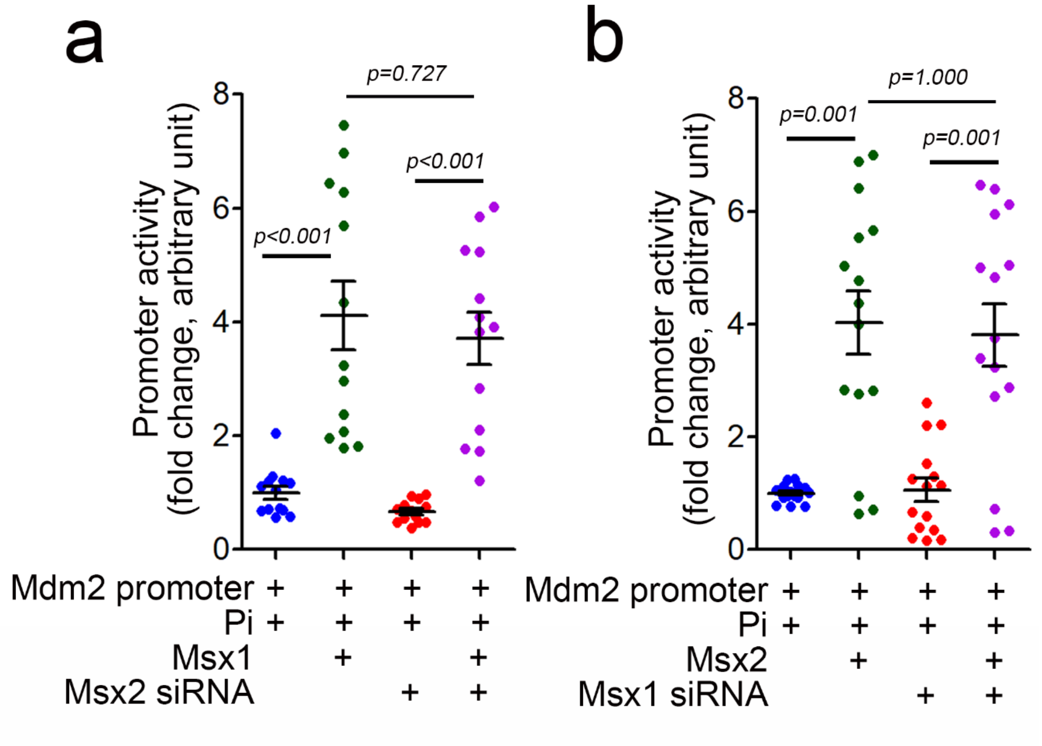

Supplementary Fig. 10. Interaction between Msx1 and Msx2 on Mdm2 promoter activation. (**a**) Transfection of Msx2 siRNA did not affect the Msx1-induced potentiation of Mdm2 promoter activity in the presence of Pi. (**b**) Effect of Msx1 siRNA on Msx2-induced potentiation of Mdm2 promoter transactivation.

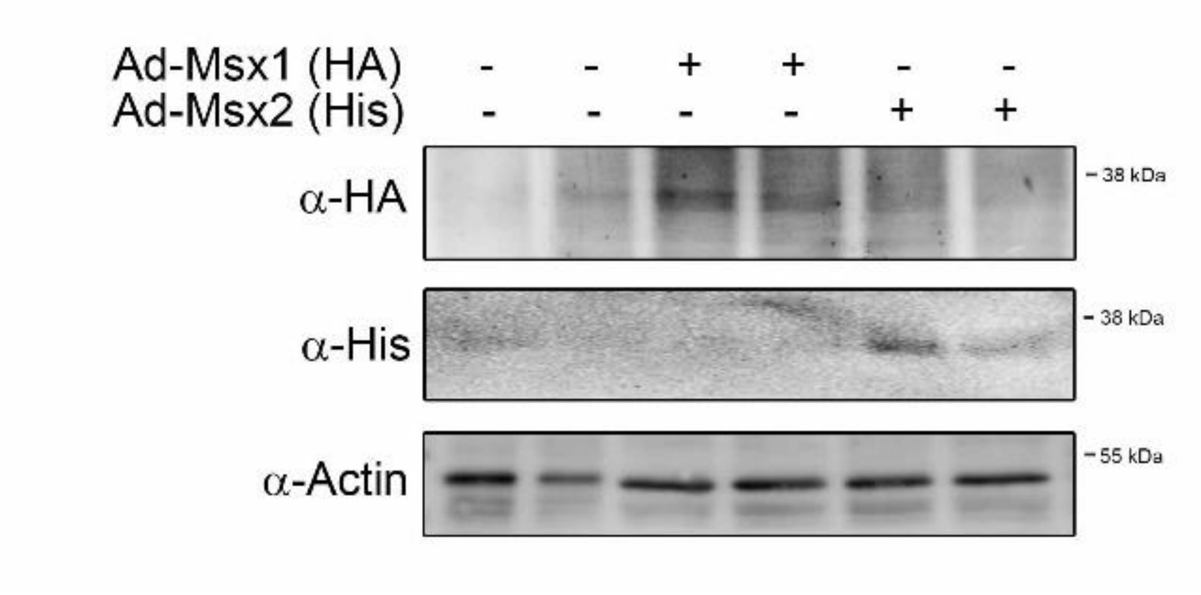

Supplementary Fig. 11. Supplemental data for adenoviral gene delivery of Msx1 and Msx2. Wild type (*Mdm2*$^{fl/fl}$) mice were subjected to tail vein injection of either Ad-Msx1-HA or Ad-Msx2-His. The expression of HA or His was examined in the aorta.

References

1 Kwon, D. H. *et al.* MDM2 E3 ligase-mediated ubiquitination and degradation of HDAC1 in vascular calcification. *Nat. Commun.* **7**, 10492 (2016).
